# An Evolutionary Trade-Off between Protein Turnover Rate and Protein Aggregation Favors a Higher Aggregation Propensity in Fast Degrading Proteins

**DOI:** 10.1371/journal.pcbi.1002090

**Published:** 2011-06-23

**Authors:** Greet De Baets, Joke Reumers, Javier Delgado Blanco, Joaquin Dopazo, Joost Schymkowitz, Frederic Rousseau

**Affiliations:** 1 VIB Switch Laboratory, VIB, Brussels, Belgium; 2 Vrije Universiteit Brussel, Brussels, Belgium; 3 Department of Bioinformatics and Genomics, Centro de Investigación Príncipe Felipe (CIPF), Valencia, Spain; 4 CIBER de Enfermedades Raras (CIBERER), Valencia, Spain; 5 Functional Genomics Node (INB) at CIPF, Valencia, Spain; Universidad de Granada, Spain

## Abstract

We previously showed the existence of selective pressure against protein aggregation by the enrichment of aggregation-opposing ‘gatekeeper’ residues at strategic places along the sequence of proteins. Here we analyzed the relationship between protein lifetime and protein aggregation by combining experimentally determined turnover rates, expression data, structural data and chaperone interaction data on a set of more than 500 proteins. We find that selective pressure on protein sequences against aggregation is not homogeneous but that short-living proteins on average have a higher aggregation propensity and fewer chaperone interactions than long-living proteins. We also find that short-living proteins are more often associated to deposition diseases. These findings suggest that the efficient degradation of high-turnover proteins is sufficient to preclude aggregation, but also that factors that inhibit proteasomal activity, such as physiological ageing, will primarily affect the aggregation of short-living proteins.

## Author Summary

In order to carry out their biological function, proteins need to fold into well-defined three-dimensional structures. Protein aggregation is a process whereby proteins misfold into inactive and often toxic higher order structures, which is implied in about 30 human diseases such as Alzheimer's disease, Parkinson's disease and systemic amyloidosis. In earlier work it has been shown that although protein aggregation is an intrinsic property of polypeptide chains that cannot be entirely avoided, evolution has optimized protein sequences to minimize the risk of aggregation in a proteome. Here we show that this pressure is not uniform, but that proteins with a short lifetime have on average a higher aggregation propensity than long-living proteins. In addition, we show that high turnover proteins also make fewer interactions with chaperones. Taken together, these observations suggest that under normal physiological conditions the aggregation propensity of short-lived proteins does not represent a significant treat for the biochemistry of the cell. Presumably the strong dependence of these proteins on proteasomal degradation is sufficient to preclude the accumulation of aggregates. As proteasomal activity declines with age this would also explain why we observe a higher association of high turnover proteins with age-dependent aggregation-related diseases.

## Introduction

Biological networks are fine-tuned to respond to narrow changes in protein concentration. The ability of a cell to maintain metabolic and signal transduction fluxes is therefore highly dependent on a tight regulation of its proteostatic network [1]. The capacity of the protein quality control system to regulate protein folding and degradation erodes with age, resulting in increased protein aggregation and aggregation-associated diseases [2], [3]. Which proteins first fall prey to misfolding is most likely a stochastic process that is modulated by both tissue-specific expression levels and environmental factors [4]. However, sensitivity to protein aggregation is also determined by intrinsic protein parameters such as the efficiency of the folding process [5], thermodynamic stability [6], [7], the aggregation propensity of the protein sequence [8], [9] and its ability to be recognized by the protein quality control system [10]. We previously showed that evolutionary forces shape protein sequences in order to minimize their aggregation propensity, by strategically placing aggregation-opposing gatekeeper residues along the sequence [11], [12]. Although this insight has been confirmed by independent studies [13], [14], [15], [16], the extent to which selective pressures mould protein sequences is most likely not uniform, but determined by the biological context in which the protein functions [17]. For instance, it has been shown that proteins with high expression levels on average have a lower aggregation propensity than proteins with lower expression levels [18]. We reasoned that proteins with high turnover rate and thus short lifetime will have, on average, lower risk of misfolding than long-living proteins. Their respective sequences should therefore also experience different selective pressures against protein aggregation. Such evolutionary pressure might have resulted in different affinities towards molecular chaperones and different implications towards aggregation-related diseases.

In order to determine the relationship between protein lifetime and protein aggregation we here combine experimental lifetime measured for 611 proteins [19] with the corresponding gene expression data in 532 healthy individuals. We also correlated experimental chaperone interaction data and structural information of these proteins to their aggregation propensity using TANGO [20], an algorithm that accurately predicts the intrinsic aggregation propensity of protein sequences. This analysis resulted in two major observations: *i)* short-living proteins on average are predicted to have longer and more severe aggregating regions than long-living proteins, and *ii)* the evolutionary enrichment of aggregation breaking gatekeeper residues is less pronounced in short-living proteins, suggesting that they experience milder selective pressure to minimize aggregation. Further, we also found significantly less interactions between short-living proteins and molecular chaperones in the IntAct database [21]. Our results suggest that under normal circumstances, protein aggregation of short-living proteins is not problematic, and thus there is little evolutionary pressure to reduce the intrinsic aggregation propensity or optimize chaperone interaction. This would turn such proteins into the Achilles' heel of the proteome in conditions where proteasomal function is significantly reduced, such as is reported for normal human ageing [22], [23], [24], [25]. In support of this hypothesis, we found that all but one of the proteins with experimentally determined turnover rates that are involved in a protein deposition disease belong to the fastest turnover rate group.

## Materials and Methods

### Scope and limitation of protein aggregation prediction

The current study focuses on short-stretch mediated protein aggregation, where specific segments of a polypeptide chain assemble into an intermolecular beta-sheet and thus nucleate aggregation. Since current knowledge in the field suggests that the short-stretch mediated protein aggregation covers the majority of disease-associated protein deposition, and no reliable prediction methods exist for alternative protein aggregation mechanisms, we feel justified to ignore alternative aggregation mechanisms such as 3D domain swapping and native protein aggregation. Like all current protein aggregation prediction algorithms, TANGO calculates intrinsic aggregation propensity of an input polypeptide sequence and returns short stretches predicted to have a high propensity to nucleate protein aggregation through the formation of intermolecular beta-sheets. These regions constitute the intrinsic aggregation propensity of the sequence in the absence of globular structure. Since these aggregation prone regions are nearly always part of the hydrophobic core when the protein resides in its native conformation, the aggregating stretches identified computationally need to become exposed by (partial) unfolding of the protein before they can actually nucleate protein aggregation. So, although three dimensional relationships that existed in the folded state are no longer relevant during assembly into an intermolecular beta-sheet, they are highly relevant to determine if a particular region is likely to become exposed in the first place. In order to estimate the likelihood that a given short polypeptide segment may become exposed by (partial) protein unfolding, we employ the FoldX force field, which calculates the contribution of each amino acid to the thermodynamic stability of the three dimensional structure of the protein, thus allowing to determine if an aggregation prone region is in a stable or less stable part of the structure.

### Datasets

#### Protein selection

Trans-membrane (TM) and extracellular proteins in the experimental dataset were excluded from the analysis. As hydrophobic trans-membrane regions of the TM proteins are not under selective pressure against aggregation they should not be considered for the analysis of the relation between protein lifetime and aggregation tendency. Since this study analyses the relation between proteasomal degradation and aggregation, extracellular proteins that are degraded by lysosomes are also deleted. We selected these proteins using the keywords “Membrane” (KW-472) and “Extracellular matrix” (KW-0272). This resulted in a dataset of 191 short-living (PSI ≤ 2) and 420 long-living (PSI ≥ 5) proteins.

#### Lifetime of proteins

Yen et al. developed a global stability analysis, a high throughput approach for proteome-scale protein-turnover analysis, resulting in a protein stability index (PSI) for 8000 human proteins [19]. PSI scores ranges from 1 to 7, with higher value indicating higher protein stability. Using a low and high cut-off value to eliminate proteins with intermediate lifetime, the dataset is split in two groups of short (PSI ≤ 2) versus long-living (PSI ≥ 5) proteins.

### Determination of aggregating sequences and flanking gatekeeper residues

The statistical mechanics algorithm TANGO [20] was used to determine the aggregation-prone regions in the human proteins. This resulted in an aggregation propensity (0–100%) for each residue, whereby an aggregating segment is defined as a continuous stretch of at least five consecutive residues, each with a TANGO score higher than 5%. The five positions before and after aggregation-prone regions are considered as “gatekeeping flanks”, with each P, R, K, E or D counting as gatekeepers [17]. No distinction was made between gatekeepers at the N or C terminus of the aggregating stretch.

### Gene expression analysis

Our dataset was composed of 532 HG-U133_Plus_2 type microarray experiments extracted from GEO (Gene Expression Omnibus) [26]. Queries were carried out using GEOmetadb module from R [27]. The dataset is composed of cancer healthy control samples only. HG-U133_Plus_2 microarrays contains probe sets of 54675 human genes per chip. All 532 chips were preprocessed in one single block using robust multichip average (RMA). RMA processing consists of three steps: background adjustment, quantile normalization and finally summarization. A list of common housekeeping genes (EIF4G2, RPL9, SFR9, GUK1, H3F3A, RHOA, ACTB) was used to confirm that the expression levels remain constant for the whole dataset. The dataset was divided into two subsets according to long-living and short-living proteins. Conversion of Affymetrix to Uniprot identifiers was done using Babelomics4 id converter [28], [29].

### FoldX modeling

Structures were selected according to the following criteria: (1) 100% sequence identity with the sequence of interest, (2) crystal structure, (3) resolution at least 3 Ä. All modeling was performed using the FoldX 2.8 force field and tool suite [30], [31]. All structures were repaired using the RepairPDB command and homology models were constructed using the BuildModel command. The stability of the aggregation nucleating regions was extracted using the SequenceDetail command.

### Statistics

Comparison of the distributions for each parameter tested in the analysis of short versus long-living proteins was performed using Mann-Whitney and Kolmogorov-Smirnov tests.

## Results

### Determination of the aggregation propensity

**Figure 1:**
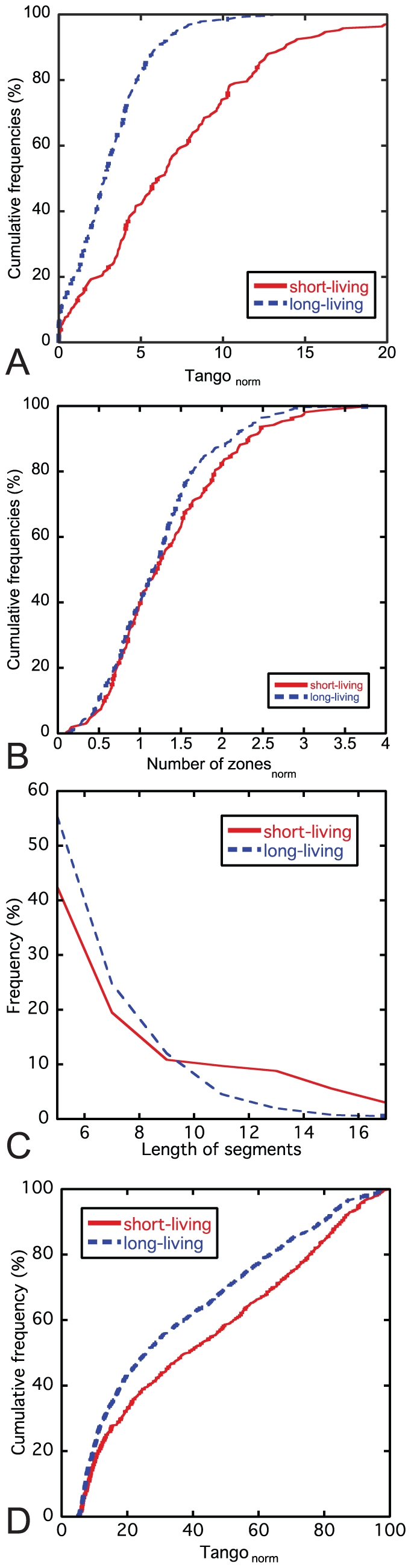
Short-living proteins display stronger aggregation propensity than long-living proteins. (A) Cumulative frequency of the length normalized TANGO scores for the short-living (PSI ≤ 2) and long-living proteins (PSI ≥ 5). The occurrence of stronger aggregating sequences (higher TANGO score) is higher in short-living proteins. (B) Cumulative frequency of the number of aggregating segments for the short-living (PSI ≤ 2) and long-living proteins (PSI ≥ 5). (C) Frequency of the length of the aggregating segments in short-living (PSI ≤ 2) and long-living proteins (PSI ≥ 5). (D) Cumulative frequency of the aggregation propensity of each aggregating segments for short-living (PSI ≤ 2) and long-living proteins (PSI ≥ 5).

**Figure 2:**
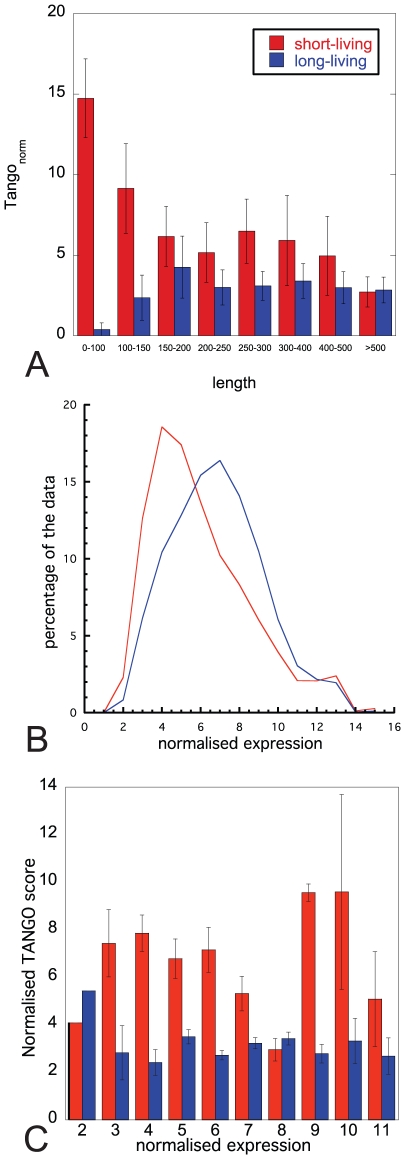
The effect of other factors that are known to modulate aggregation propensity. (A) Average aggregation propensity in function of the protein length for short-living (PSI ≤ 2) and long-living (PSI ≥ 5) proteins. (B) Density plot of the normalized expression level of the proteins from our short-living and long-living groups recorded from microarray data from 532 healthy human individuals. (C) Difference in aggregation load between fast and slow turnover proteins separated by expression level.

Yen et al. developed a global stability analysis, a high throughput approach for proteome-scale protein-turnover analysis, resulting in a protein stability index (PSI) for 8000 human proteins [19]. PSI scores ranges from 1 to 7, with higher values indicating higher biological protein stability and thus slower protein turnover. To simplify the analysis, we used a low and a high cut-off value to eliminate proteins with intermediate lifetime, so that the data were split in two groups of short (PSI ≤ 2) versus long-living (PSI ≥ 5) proteins (Text S1). A number of characteristics of the aggregation propensity of these 611 proteins were determined using the TANGO algorithm [20]: *i)* the average aggregation propensity of the protein (total TANGO score normalized by protein length), *ii)* the number of aggregating segments in the protein, *iii)* the length of aggregating segments, and *iv)* the aggregation propensity of each aggregating segment. The correlation with the experimentally determined biological lifetime of the protein was tested for each individual parameter and significant differences were found (Text S1): Short-living proteins display a higher average aggregation propensity (Figure 1A), which is not caused by an increase in the average number of aggregating segments (Figure 1B), but by an significant increase in their length (Figure 1C) and aggregation propensity (Figure 1D). As previous studies have shown that long proteins on average have less effective aggregation-promoting regions than shorter proteins [32] and the average length of short and long-living proteins is respectively 263 and 357 amino acids, the aforementioned observations could also be due to the longer mean length of long-living proteins. In order to exclude this possibility, we repeated the analysis after the exclusion of proteins longer than 300 amino acids, and found that the difference in aggregation tendency between the two lifetime categories remains significant (p<0.001), showing that the observed difference in aggregation tendency is linked to the disparity in lifetime, and is independent of the difference in mean length of the proteins. This conclusion is confirmed by plotting the average aggregation tendency in function of the protein length for each lifetime category (Figure 2A). In view of the idea introduced by Vendrusculo and co-workers that protein expression levels are tuned to the solubility limit of the protein [18], we need to exclude that the difference in aggregation load in our data is simply due to a lower expression level for the fast turnover proteins. To address this, we employed publically available microarray data from the Gene Expression Omnibus (GEO) [33], corresponding to 532 healthy individuals from 62 studies to compare expression levels of the proteins in our lifetime dataset. The density plot of the normalized expression levels for all proteins from the short lifetime and long lifetime groups reveals indeed a different composition of both groups in terms of expression levels (Figure 2B). However, when we plot the length normalized aggregation score of the short and long-living proteins grouped per expression level (Figure 2C), we see that the expression level is not the determining factor in the difference in aggregation propensity between fast and slow turnover proteins. These results suggest that proteins with a short biological lifetime undergo less evolutionary pressure to minimize the burden of aggregation.

### The influence of thermodynamic stability of the protein

**Figure 3:**
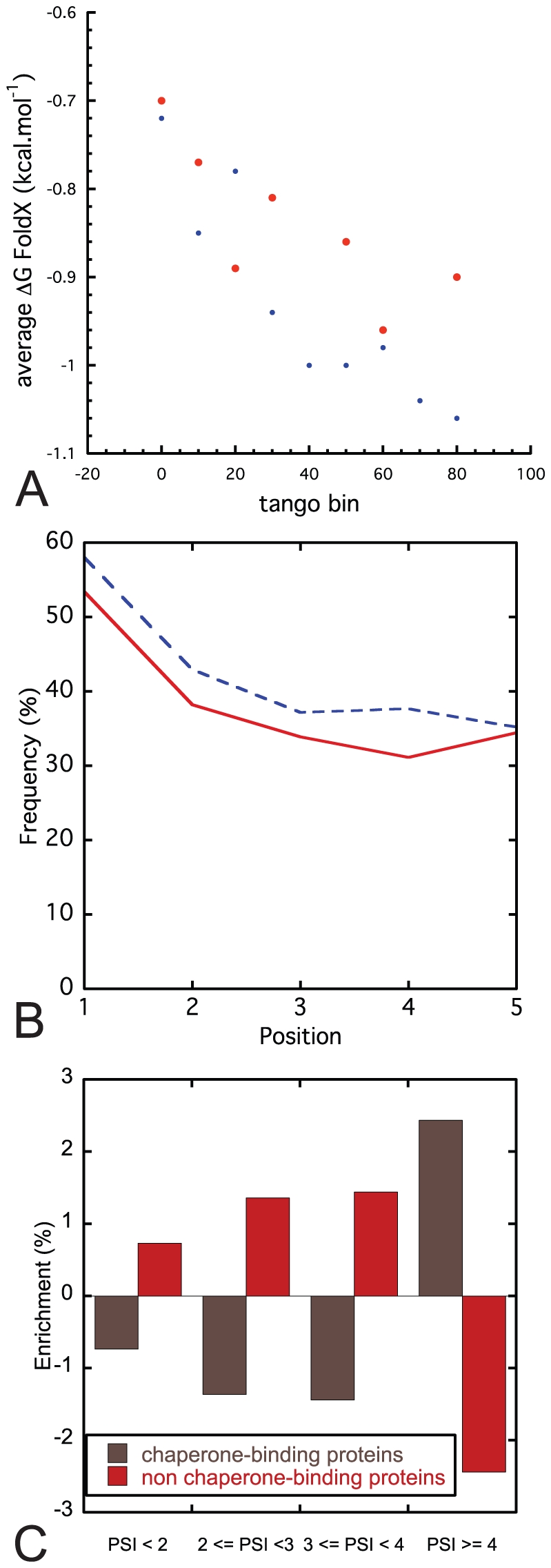
The effect of cellular and evolutionary mechanisms that counteract protein aggregation. (A) A plot of the average thermodynamic stability of aggregation nucleating regions in the context of a native folded protein in function of the aggregation propensity shows that the most strongly aggregating segments are on average buried in the most stable regions of the protein and that this trend is similar for the long and short-living proteins in our set. The correlation values on the raw unaveraged data are 0.43 and 0.30 for short and long-living protein respectively, which rise to 0.79 and 0.91 in the bin-average plot shown here. (B) Frequency of the gatekeepers (residues P, R, K, D, E) for the short-living (PSI ≤ 2) and long-living proteins (PSI ≥ 5). (C) Enrichment of the chaperone-binding and non-chaperone-binding proteins for the different lifetime categories.

An alternative explanation for the lower sensitivity of fast turnover proteins to the evolutionary pressure against protein aggregation could be that these proteins possess native structures with inherently superior thermodynamic stability to those of proteins from the long lifetime group. Given the significant structural coverage of our dataset, i.e. there are high resolution crystallographic structures available for 127 proteins in our dataset of 611 (Text S1), we can address this question using a modeling approach. To do so we employed the FoldX force field [31] to calculate the thermodynamic stability of the aggregation nucleating regions predicted by TANGO in the corresponding crystal structures. We then plotted the average thermodynamic stability of the aggregating nucleating regions per bin of aggregation propensity according to TANGO (Figure 3A). In this plot, we observe a clear correlation between the aggregation propensity of a polypeptide stretch and thermodynamic stability of the same region in the context of its native three-dimensional structure, so that sequences with the highest aggregation propensity form the most stable parts of the protein structure under native conditions, which is in accordance with previous observations [5]. Importantly, Figure 3A reveals no significant differences between proteins with a long or a short lifetime, showing that the difference in aggregation propensity between these groups is not due to fundamental differences in protein architecture or thermodynamic stability.

### Occurrence of gatekeeper residues to oppose protein aggregation

It has been well established that evolutionary pressure against protein aggregation has resulted in the enrichment at the flanks of aggregation prone segments of *gatekeeper* residues, a term used to indicate amino acids that counteract aggregation [12], [15], [34]. This disruption of the aggregation prone stretches is achieved by a) the repulsive effect of charge (arginine, aspartate, glutamate), b) the entropic penalty for burial (arginine and lysine) or c) incompatibility with beta-structure conformation (proline) [34]. We analyzed the frequency of occurrence of gatekeeper residues in our short- and long-living protein datasets and found that the frequency of occurrence of gatekeeper residues shows a small but significant reduction in short-living proteins (Figure 3B), which indicates that the introduction of gatekeepers as an evolutionary mechanism, to minimize aggregation is less pronounced in this set. This is consistent with the observation of longer aggregating stretches since they are less frequently interrupted by aggregation breaking residues, resulting also in a higher aggregation propensity of the stretches.

### Relation between protein stability and chaperone binding

A major component of the protein quality control system that evolved in all forms of cellular life to deal with the unavoidable burden of protein misfolding and aggregation is formed by the diverse families of molecular chaperones, which are a class of proteins that assist other proteins in (re)folding and disaggregation and eventually shuttle substrates to the degradation machinery [35]. In order to address the question if protein turnover rates influence the requirement of chaperone assistance of a protein, we searched the protein interaction database IntAct [21] (release March 19, 2010) for experimentally recorded interactions between proteins from our dataset and an extensive list of known human molecular chaperones (listed in Text S1). A total of 237 chaperone-binding proteins were identified, but experimentally determined protein stability was available for only 114 proteins. Based on Yen *et al.*, we divided this set of proteins into four categories according to their PSI turnover scores: short half-life (PSI < 2), medium half-life (2 ≤ PSI<3), long half-life (3 ≤ PSI<4) and extra-long half-life (PSI ≥ 4) [19]. For each category we calculated the enrichment of chaperone-binding proteins, where enrichment is defined as *PSI_N_/PSI_T_ – SUM_N_/SUM_T_*. PSI_x_ is the number of proteins in a given set x, belonging to a given PSI category and SUM_x_ the total number of proteins in a given set x. X points to the total set (T) or the (non-) chaperone-binding proteins (N). Comparison of the chaperone enrichment in short-living versus long-living proteins shows that in our limited dataset, proteins that interact with molecular chaperones are significantly enriched in the group of long-living proteins (Figure 3C). Given we observed no fundamental differences in the thermodynamic stability or protein architecture between these groups (see FoldX analysis above), this suggests that short-living proteins on average require less chaperone intervention than long-living proteins, consistent with the notion that their fast degradation rate is sufficient to protect against misfolding and aggregation.

### Relation between disease-associated mutations and lifetime

We investigated which of the proteins in our dataset are involved in a human disease associated with protein deposition and found 16 proteins with known PSI score (Text S1). Interestingly, all but one of these proteins belong to the category of short (PSI < 2) or medium (2 ≤ PSI < 3) half-life. Although this analysis is not exhaustive, the data does suggest that the lack of evolutionary pressure to reduce aggregation in short-living proteins can backfire in circumstances were their turnover is altered.

## Discussion

**Figure 4:**
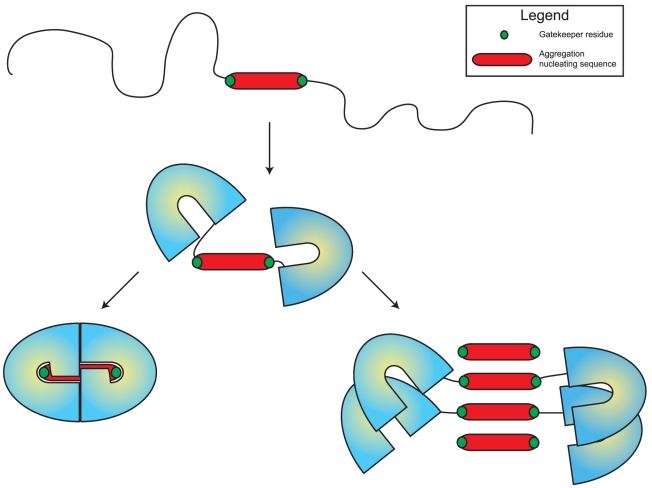
Schematic representation of the short-stretch hypothesis of protein aggregation. In the unfolded state (top) and partially folded intermediates, the protein exposes an aggregation nucleating stretch, that becomes buried upon folding into the globular native structure (bottom left). In a competing reaction, aggregation-prone stretches may align into an intermolecular β-sheet, effectively nucleating the formation of a protein aggregate (bottom right). The gatekeeper residues indicated in green reduce the rate of the aggregation reaction by interfering with the beta-sheet structure through steric hindrance and charge repulsion.

Protein aggregation is triggered by short polypeptide stretches within a protein sequence that assemble into intermolecular beta-sheets when they become exposed to the solvent [8], [36], [37] (Figure 4). These aggregation nucleating regions can be predicted with good accuracy with biocomputational tools [20], [38], [39], [40], [41], [42], [43], [44], [45], [46], [47], [48], [49], [50], [51] and earlier work has shown that their occurrence is an inevitable consequence of the structural requirements of protein structure [52]. Globular protein architecture requires the tertiary packing of hydrophobic secondary structure elements to form a stable hydrophobic core. Unfortunately, these physicochemical parameters are also associated to a high probability for self-assembly of such secondary structure elements into β-aggregates [53], [54]. Indeed, less than 10% of globular protein domains are devoid of aggregation propensity [12]. As a consequence of these overlapping but opposing forces that govern protein folding and aggregation, protein folding is generally a very inefficient process [55], [56]. Moreover, aggregation is detrimental for the cell as misfolded proteins are inactive [57] and can acquire toxic gain-of-function [58]. Protein homeostasis is therefore tightly regulated by the protein quality control machinery of the cell.

Given the high burden of protein aggregation on the proteome, and even if aggregation propensity cannot be avoided altogether, selective pressure to minimize the aggregation propensity of protein sequences is still to be expected. Indeed, it was found that aggregation-opposing residues are enriched at specific sites along the sequence of proteins [12], [59]. These so-called aggregation-gatekeepers residues, consisting of prolines and charged amino acids, are systematically found at the flanks of aggregation-prone sequences stretches within proteins. Due to their β-breaking nature or charge they efficiently lower the aggregation propensity of hydrophobic stretches while at the same time preserving hydrophobic cores by their peripheral placement (Figure 4). Removal of gatekeepers increases aggregation and as a result gatekeeper mutations are three times more frequent in human disease mutants than in human polymorphisms [17], [60].

Selective pressure against aggregation is not homogeneous. We previously showed that enrichment of gatekeeper residues is more pronounced at the flanks of strongly aggregating sequences [12] and it was also shown that aggregation propensity inversely correlates with gene expression [18]. In this study we employed the TANGO aggregation prediction tool [20] to compare the aggregation characteristics of proteins taken from the extremes of the protein lifetime distribution from the large scale data by Yen *et al* [19]. We observe a significantly higher aggregation propensity in proteins with a short lifetime than in proteins with a long lifetime. Analysis of gene expression data in 532 healthy individuals excluded the possibility that the observed difference in aggregation propensity arises from differences in gene expression levels between short-living and long-living proteins. Additionally the FoldX [31] analysis of the structures from both groups of proteins clearly show that this is not a result from a superior thermodynamic stability of short lifetime proteins, but rather from a genuinely higher aggregation propensity of their protein sequence. The higher aggregation propensity of short-living proteins does not originate from a higher number of aggregating regions, but rather from the higher average length and aggregation propensity of these regions, which can be traced back to a reduction in the amount of aggregation breaking gatekeeper residues. Hence, the reduced placement of gatekeepers in short-living proteins and the resulting higher average aggregation propensity, is evidence for the fact that proteins with a fast turnover rate experience less selective pressure to minimize aggregation than proteins with a longer biological lifetime.

Moreover, a search of the IntAct database [21] revealed that there are significantly more recorded chaperone interactions for long-living proteins than short-living proteins. So, not only do short-living proteins experience milder selective pressure against aggregation, but at the same time they also interact less frequently with molecular chaperones or at least form less stable interactions of the type that can be recorded by current experimental techniques. Taken together, these data strongly suggest that the misfolding of short-living proteins is generally not affecting the fitness of the cell, as presumably the strong dependence of these proteins on proteasomal degradation suffices to avoid the accumulation of protein aggregates.

On the other hand, it is known that the efficiency of the proteasomal system erodes as a result of physiological ageing [61], [62], [63]. Under these changing conditions, proteins with a higher aggregation propensity and lacking sufficient affinity for chaperones would form the Achilles' heel of the proteome and be among the most susceptible to aggregate. In this respect it is interesting to see that some of the fast turnover proteins from the dataset are indeed associated with human diseases with a protein deposition phenotype.

## Supporting Information

Text S1 **Supplementary data. Table 1. Comparison between the aggregation parameters for short-living and long-living proteins.** The analysed population is the group of short-living protein, the reference population are the long-living proteins. ++ and − indicate that the population has a distribution significantly (p<0.001) shifted to respectively higher or lower values than the reference population in the performed statistical test, idem for + and − where p<0.01. **Table 2. Lifetime data for disease-associated proteins.** We show the lifetime values of the proteins from the Yen dataset [19] on protein lifetime that are associated with protein deposition diseases. **Table 3. Overview of the protein set used.** From the Yen dataset [19] on protein lifetime, we here show the lifetime values for the 611 proteins that fall in the extreme categories (longest and shortest lifetimes respectively). Where high resolution structural information is available in the Protein Structure Databank (PDB) (http://www.pdb.org) [64] we indicate the PDBID. **Table 4. Overview of the chaperone set.** This table contains the chaperones used in the IntAct [21] interaction study, represented by their accession number, entry name and UniProt comment.
(DOC)
