# Supplementary material for: An Evolutionary Trade-Off between Protein Turnover Rate and Protein Aggregation Favors a Higher Aggregation Propensity in Fast Degrading Proteins
Source: PLoS Comput Biol. 2011 Jun 23;7(6):e1002090. doi: 10.1371/journal.pcbi.1002090 (PMC3121684; doi:10.1371/journal.pcbi.1002090)
Supplement: Text S1 — Supplementary data. Table 1. Comparison between the aggregation parameters for short-living and long-living proteins. The analysed population is the group of short-living protein, the reference population are the long-living proteins. ++ and − indicate that the population has a distribution significantly (p<0.001) shifted to respectively higher or lower values than the reference population in the performed statistical test, idem for + and − where p<0.01. Table 2. Lifetime data for disease-associated proteins. We show the lifetime values of the proteins from the Yen dataset [19] on protein lifetime that are associated with protein deposition diseases. Table 3. Overview of the protein set used. From the Yen dataset [19] on protein lifetime, we here show the lifetime values for the 611 proteins that fall in the extreme categories (longest and shortest lifetimes respectively). Where high resolution structural information is available in the Protein Structure Databank (PDB) (http://www.pdb.org) [64] we indicate the PDBID. Table 4. Overview of the chaperone set. This table contains the chaperones used in the IntAct [21] interaction study, represented by their accession number, entry name and UniProt comment. (DOC) [file pcbi.1002090.s001.doc]

**Supplementary data**

**Table 1:** **Comparison between the aggregation parameters for short-living and long-living proteins.** The analysed population is the group of short-living protein, the reference population are the long-living proteins. ++ and – indicate that the population has a distribution significantly (p < 0.001) shifted to respectively higher or lower values than the reference population in the performed statistical test, idem for + and – where p < 0.01.

|  | Kolmogorov-Smirnov | Mann-Whitney test |
| --- | --- | --- |
| Average aggregation propensity (total Tango/length) | ++ | ++ |
| Number of aggregating segments | -- | -- |
| Number of aggregating segments/length | n.s. | n.s. |
| Length of aggregating segments | ++ | ++ |
| Aggregation propensity of aggregating segments | ++ | ++ |

**Table 2: Lifetime data for disease-associated proteins.** We show the lifetime values of the proteins from the Yen dataset [1] on protein lifetime that are associated with protein deposition diseases.

| Protein Name | PSI score | Aggregation propensity |
| --- | --- | --- |
| Amyloid beta A4 precursor protein^a^ | 2.84 | 3.54 |
| Apolipoprotein A-I | 2.34 | 5.13 |
| Atrial natriuretic factor | 2.20 | 7.37 |
| Insulin | 1.56 | 7.43 |
| Lithostathine-1-alpha | 2.27 | 10.9 |
| Lysozyme C | 1.96 | 8.37 |
| Lactadherin | 2.84 | 1.43 |
| Major prion protein | 2.21 | 9.60 |
| Serum amyloid A protein | 1.79 | 10.74 |
| Superoxide dismutase | 5.6 | 0.61 |
| Transthyretin | 2.15 | 10.02 |
| Lactotransferrin | 2.62 | 5.25 |
| Glucagon | 1.98 | 12.21 |
| Transforming growth factor-beta-induced protein ig-h3 | 1.96 | 3.11 |
| Apolipoprotein A-II | 1.64 | 15.83 |
| TAR DNA-binding protein 43 | 2.11 | 4.02 |

^a^ The clearance rate of the Abeta peptide in blood is even higher with a reported half life of approximately 5 mins [2].

**Table 3:** **Overview of the protein set used.** From the Yen dataset [1] on protein lifetime, we here show the lifetime values for the 611 proteins that fall in the extreme categories (longest and shortest lifetimes respectively). Where high resolution structural information is available in the Protein Structure Databank (PDB) ([http://www.pdb.org](http://www.pdb.org/)) [3] we indicate the PDBID.

| **gene_name** | **PSI** | **PDBID** |
| --- | --- | --- |
| 1433E_HUMAN | 6.49 | 2br9 |
| 1433G_HUMAN | 6.09 |  |
| 1433S_HUMAN | 5.88 | 1yz5 |
| 2AAA_HUMAN | 5.16 | 3k7w |
| 2B32_HUMAN | 1.85 |  |
| 2B51_HUMAN | 1.86 | 1zgl |
| 2DMB_HUMAN | 1.64 | 2bc4 |
| 2DOB_HUMAN | 1.98 |  |
| 3MG_HUMAN | 5.08 |  |
| 6PGD_HUMAN | 5.68 |  |
| ACO11_HUMAN | 1.88 |  |
| ACSF4_HUMAN | 1.60 |  |
| ADRM1_HUMAN | 1.84 |  |
| AER61_HUMAN | 1.72 |  |
| AMY2B_HUMAN | 1.65 |  |
| AMYP_HUMAN | 1.59 | 1b2y |
| ANFB_HUMAN | 1.78 | 1yk1 |
| ANGL7_HUMAN | 1.75 |  |
| ANKR1_HUMAN | 1.89 |  |
| ANR22_HUMAN | 1.77 |  |
| APOA2_HUMAN | 1.64 | 2ou1 |
| APOC2_HUMAN | 1.75 | 1soh |
| APOC3_HUMAN | 1.80 |  |
| APOL1_HUMAN | 1.50 |  |
| APOL4_HUMAN | 2.00 |  |
| ARH_HUMAN | 5.04 | 2g30 |
| ASPX_HUMAN | 1.76 |  |
| B2LA1_HUMAN | 1.62 | 3i1h |
| B2MG_HUMAN | 1.59 | 3kyo |
| C1QT6_HUMAN | 1.84 |  |
| CA109_HUMAN | 1.91 |  |
| CA128_HUMAN | 1.97 |  |
| CAH6_HUMAN | 1.75 |  |
| CAPZB_HUMAN | 1.66 |  |
| CATB_HUMAN | 1.78 | 3pbh |
| CCKN_HUMAN | 1.36 |  |
| CCL21_HUMAN | 1.92 |  |
| CG034_HUMAN | 1.98 |  |
| CH004_HUMAN | 1.99 |  |
| CH059_HUMAN | 1.72 |  |
| CHD9_HUMAN | 1.37 |  |
| CHP1_HUMAN | 2.00 | 2e30 |
| CIDEA_HUMAN | 1.89 | 2eel |
| CIDEC_HUMAN | 1.77 |  |
| CMTA2_HUMAN | 1.94 |  |
| CN128_HUMAN | 1.69 |  |
| CNOT7_HUMAN | 1.83 | 2d5r |
| COAC_HUMAN | 5.49 |  |
| CP052_HUMAN | 1.62 |  |
| CPSF5_HUMAN | 5.28 | 3bap |
| CRIS2_HUMAN | 1.88 |  |
| CSH_HUMAN | 1.77 |  |
| CT024_HUMAN | 1.73 |  |
| CTRB1_HUMAN | 1.76 |  |
| CXCL6_HUMAN | 1.58 |  |
| CXL11_HUMAN | 1.60 | 1rjt |
| CYHR1_HUMAN | 1.60 |  |
| CYTF_HUMAN | 1.61 | 2ch9 |
| DCUP_HUMAN | 5.64 | 3gvr |
| DEF1_HUMAN | 1.55 | 3hjd |
| DEXI_HUMAN | 1.36 |  |
| DHPR_HUMAN | 6.15 |  |
| DHRS7_HUMAN | 1.91 |  |
| DHX37_HUMAN | 1.97 |  |
| DJB11_HUMAN | 1.64 |  |
| DJC10_HUMAN | 1.93 |  |
| DNJB9_HUMAN | 1.58 |  |
| DNJC3_HUMAN | 1.55 |  |
| DPH5_HUMAN | 1.70 |  |
| DPYD_HUMAN | 1.85 |  |
| DSCR8_HUMAN | 1.74 |  |
| DUS22_HUMAN | 1.88 | 1wrm |
| DYDC1_HUMAN | 1.76 |  |
| EGLN1_HUMAN | 1.55 | 3hqu |
| ELAF_HUMAN | 1.85 | 2rel |
| EMAL4_HUMAN | 1.91 |  |
| F10A1_HUMAN | 1.55 |  |
| F19A4_HUMAN | 1.60 |  |
| FAM3A_HUMAN | 1.79 |  |
| FBX15_HUMAN | 1.94 |  |
| FIBG_HUMAN | 1.73 | 2a45 |
| FKBP7_HUMAN | 1.73 |  |
| FOSL2_HUMAN | 2.00 |  |
| GAGC1_HUMAN | 5.23 |  |
| GALA_HUMAN | 1.71 | 1smz |
| GATA2_HUMAN | 1.53 |  |
| GGEE1_HUMAN | 5.42 |  |
| GHRL_HUMAN | 1.66 |  |
| GLHA_HUMAN | 1.85 | 1xwd |
| GLUC_HUMAN | 1.98 | 3iol |
| GLYC_HUMAN | 5.11 |  |
| GP175_HUMAN | 1.58 |  |
| GRAB_HUMAN | 1.76 | 1fq3 |
| HA2Q_HUMAN | 1.81 |  |
| HB25_HUMAN | 1.91 |  |
| HB2B_HUMAN | 1.82 | 1a6a |
| HB2X_HUMAN | 1.91 |  |
| HES2_HUMAN | 1.70 |  |
| HIS3_HUMAN | 1.77 |  |
| HMGB4_HUMAN | 1.90 |  |
| HNMT_HUMAN | 1.93 | 2aox |
| HNRPG_HUMAN | 5.33 |  |
| IGF2_HUMAN | 1.80 |  |
| IL15_HUMAN | 1.57 |  |
| IL32_HUMAN | 1.66 |  |
| ILEU_HUMAN | 5.36 |  |
| ILF2_HUMAN | 5.58 |  |
| ILKAP_HUMAN | 5.92 |  |
| IMDH2_HUMAN | 6.18 | 1nfb |
| IMPA1_HUMAN | 5.22 | 1imf |
| ING3_HUMAN | 1.97 | 1x4i |
| ING5_HUMAN | 1.99 | 3c6w |
| INO1_HUMAN | 5.02 |  |
| INS_HUMAN | 1.56 | 5aiy |
| IPP2_HUMAN | 5.45 |  |
| ISK1_HUMAN | 1.77 |  |
| ISK2_HUMAN | 1.39 | 2jxd |
| ISLR_HUMAN | 1.66 |  |
| K1609_HUMAN | 5.12 |  |
| K1C23_HUMAN | 5.99 |  |
| K6PF_HUMAN | 5.50 |  |
| K6PP_HUMAN | 6.04 |  |
| KATL2_HUMAN | 5.63 |  |
| KHDR2_HUMAN | 5.08 |  |
| KHDR3_HUMAN | 5.35 |  |
| KIME_HUMAN | 6.04 |  |
| KLK1_HUMAN | 1.71 |  |
| KLK10_HUMAN | 1.64 |  |
| KPCZ_HUMAN | 5.34 |  |
| KPYM_HUMAN | 5.03 | 3h6o |
| KSYK_HUMAN | 6.28 | 3fqs |
| LACB2_HUMAN | 5.32 |  |
| LBH_HUMAN | 1.79 |  |
| LC7L2_HUMAN | 5.29 |  |
| LCHN_HUMAN | 1.68 |  |
| LDOC1_HUMAN | 1.61 |  |
| LEG3_HUMAN | 5.04 | 2nn8 |
| LEGL_HUMAN | 1.54 | 2jj6 |
| LICH_HUMAN | 1.72 |  |
| LIPR1_HUMAN | 1.88 |  |
| LMCD1_HUMAN | 5.76 |  |
| LMNA_HUMAN | 5.09 | 1ivt |
| LPXN_HUMAN | 5.14 |  |
| LRC28_HUMAN | 1.94 |  |
| LRSM1_HUMAN | 6.10 |  |
| LXN_HUMAN | 5.88 |  |
| LYPA1_HUMAN | 5.07 | 1fj2 |
| LYPA2_HUMAN | 5.35 |  |
| LYSC_HUMAN | 1.96 | 1c46 |
| LZTL1_HUMAN | 5.68 |  |
| M3K3_HUMAN | 1.73 | 2o2v |
| M6PBP_HUMAN | 6.35 |  |
| MAGA8_HUMAN | 5.11 |  |
| MAGA9_HUMAN | 5.01 |  |
| MARE2_HUMAN | 5.94 |  |
| MATK_HUMAN | 1.67 | 1jwo |
| MCH_HUMAN | 1.76 |  |
| MDM1_HUMAN | 1.75 |  |
| MGLL_HUMAN | 5.76 |  |
| MGMT_HUMAN | 5.58 |  |
| MIA_HUMAN | 1.70 | 1k0x |
| MINA_HUMAN | 5.05 |  |
| MK_HUMAN | 1.74 | 1mkn |
| MK12_HUMAN | 5.81 | 1cm8 |
| MKNK2_HUMAN | 1.86 |  |
| MNDA_HUMAN | 6.21 | 2dbg |
| MP2K2_HUMAN | 5.36 |  |
| MP2K3_HUMAN | 5.60 |  |
| MP2K6_HUMAN | 5.96 |  |
| MPPD2_HUMAN | 5.16 |  |
| MRE11_HUMAN | 1.92 |  |
| MRP_HUMAN | 6.34 |  |
| MT1E_HUMAN | 5.39 |  |
| MT1H_HUMAN | 5.39 |  |
| MT1M_HUMAN | 5.33 |  |
| MT1X_HUMAN | 5.44 |  |
| MT3_HUMAN | 5.48 |  |
| MTF1_HUMAN | 5.40 |  |
| MTG8_HUMAN | 5.32 | 2knh |
| MTHFS_HUMAN | 5.14 |  |
| MYCT1_HUMAN | 1.70 |  |
| MYEOV_HUMAN | 1.97 |  |
| MYOME_HUMAN | 1.94 |  |
| NADK_HUMAN | 5.61 |  |
| NASP_HUMAN | 5.65 |  |
| NCALD_HUMAN | 5.12 | 1bjf |
| NCK2_HUMAN | 5.66 | 1u5s |
| NDKA_HUMAN | 5.64 | 2hvd |
| NDRG4_HUMAN | 5.02 |  |
| NEIL1_HUMAN | 5.46 |  |
| NF2L2_HUMAN | 2.00 | 2flu |
| NMB_HUMAN | 1.98 | 1c9a |
| NNAT_HUMAN | 1.71 |  |
| NOE1_HUMAN | 1.95 |  |
| NP1L1_HUMAN | 5.50 |  |
| NPC2_HUMAN | 1.53 |  |
| NPY_HUMAN | 1.63 | 1ron |
| NRF1_HUMAN | 5.28 |  |
| NSF1C_HUMAN | 5.18 | 1ss6 |
| NTF2_HUMAN | 5.62 | 1oun |
| NTKL_HUMAN | 5.11 |  |
| NUD11_HUMAN | 5.45 |  |
| NUDC_HUMAN | 5.17 |  |
| NUDT3_HUMAN | 5.09 |  |
| NXF1_HUMAN | 5.36 | 1oai |
| OCAD2_HUMAN | 1.92 |  |
| OGT1_HUMAN | 5.38 |  |
| OXSR1_HUMAN | 5.01 | 2vwi |
| P2R3B_HUMAN | 1.89 |  |
| P53_HUMAN | 5.11 | 2ocj |
| P5CR3_HUMAN | 5.38 |  |
| PA1B2_HUMAN | 5.30 | 1vyh |
| PA1B3_HUMAN | 5.60 |  |
| PA21B_HUMAN | 1.48 |  |
| PA2GD_HUMAN | 1.61 |  |
| PACN1_HUMAN | 5.78 |  |
| PACN2_HUMAN | 5.15 |  |
| PAK7_HUMAN | 5.20 |  |
| PANK2_HUMAN | 1.84 |  |
| PAPS2_HUMAN | 5.31 | 2ax4 |
| PCGF3_HUMAN | 2.00 |  |
| PCY2_HUMAN | 5.31 | 3elb |
| PDCD5_HUMAN | 5.02 | 2cru |
| PDCL2_HUMAN | 5.46 |  |
| PDCL3_HUMAN | 5.65 |  |
| PDE1B_HUMAN | 6.14 |  |
| PDLI1_HUMAN | 5.11 |  |
| PDYN_HUMAN | 1.95 |  |
| PENK_HUMAN | 1.82 |  |
| PEPD_HUMAN | 6.04 |  |
| PFD2_HUMAN | 5.17 |  |
| PFD4_HUMAN | 5.35 |  |
| PG12A_HUMAN | 1.92 |  |
| PGAM2_HUMAN | 6.36 |  |
| PGM1_HUMAN | 6.04 |  |
| PIP_HUMAN | 1.91 | 3es6 |
| PIPNB_HUMAN | 5.08 |  |
| PMM2_HUMAN | 5.55 | 2q4r |
| PP1R8_HUMAN | 5.16 |  |
| PP2BA_HUMAN | 5.31 | 1aui |
| PPCT_HUMAN | 5.14 | 1ln3 |
| PPIL1_HUMAN | 5.09 |  |
| PPIP1_HUMAN | 5.92 |  |
| PPM1A_HUMAN | 5.97 |  |
| PPM1G_HUMAN | 5.13 |  |
| PPR1A_HUMAN | 5.82 |  |
| PPR1B_HUMAN | 5.69 |  |
| PROF1_HUMAN | 5.55 |  |
| PROSC_HUMAN | 5.53 |  |
| PRP4_HUMAN | 5.23 |  |
| PRPS1_HUMAN | 5.65 | 3efh |
| PRS7_HUMAN | 5.23 |  |
| PSD10_HUMAN | 5.58 | 1uoh |
| PSME2_HUMAN | 5.14 |  |
| PSPC_HUMAN | 1.84 |  |
| PTBP2_HUMAN | 6.09 | 2cq1 |
| PTN7_HUMAN | 5.27 |  |
| PUR6_HUMAN | 6.06 | 2h31 |
| PURA2_HUMAN | 1.49 | 2v40 |
| PYGB_HUMAN | 5.44 |  |
| R3GEF_HUMAN | 5.36 |  |
| RAB7B_HUMAN | 1.94 |  |
| RAF1_HUMAN | 5.57 | 3lb7 |
| RAN_HUMAN | 5.60 | 3ch5 |
| RB15B_HUMAN | 5.02 |  |
| RBBP9_HUMAN | 5.54 |  |
| RBM3_HUMAN | 5.28 |  |
| RBM5_HUMAN | 5.34 |  |
| RBMS1_HUMAN | 5.05 |  |
| RCC1_HUMAN | 5.68 |  |
| REG3A_HUMAN | 1.78 | 1uv0 |
| RFA2_HUMAN | 5.38 | 2pqa |
| RFA3_HUMAN | 1.31 | 2z6k |
| RIFK_HUMAN | 6.12 |  |
| RIR1_HUMAN | 5.13 | 2wgh |
| RL10L_HUMAN | 1.68 |  |
| RL13A_HUMAN | 1.95 |  |
| RL23_HUMAN | 1.78 |  |
| RL23A_HUMAN | 1.69 | 2zkr |
| RL27_HUMAN | 1.63 |  |
| RL28_HUMAN | 1.83 |  |
| RL30_HUMAN | 5.40 | 2zkr |
| RL5_HUMAN | 1.76 |  |
| RL8_HUMAN | 1.59 |  |
| RLA1_HUMAN | 5.03 | 2jdl |
| RLBP1_HUMAN | 5.66 |  |
| RM33_HUMAN | 1.92 | 3iy9 |
| RM42_HUMAN | 1.96 |  |
| RN111_HUMAN | 1.49 |  |
| RNF26_HUMAN | 1.91 |  |
| RNF32_HUMAN | 1.72 |  |
| RNPS1_HUMAN | 5.42 |  |
| RNS11_HUMAN | 1.62 |  |
| RNT2_HUMAN | 1.77 |  |
| ROP1B_HUMAN | 1.93 |  |
| RPE_HUMAN | 5.26 |  |
| RPIA_HUMAN | 5.21 |  |
| RS12_HUMAN | 5.48 |  |
| RS4Y1_HUMAN | 1.91 |  |
| RSPO2_HUMAN | 1.77 |  |
| RUSD2_HUMAN | 5.52 |  |
| RWDD1_HUMAN | 5.21 | 2ebm |
| S10A7_HUMAN | 5.00 |  |
| SAA_HUMAN | 1.79 |  |
| SAPS3_HUMAN | 6.47 |  |
| SCLY_HUMAN | 6.36 |  |
| SCRG1_HUMAN | 1.66 |  |
| SDCG3_HUMAN | 5.29 |  |
| SDSL_HUMAN | 5.20 |  |
| SELT_HUMAN | 1.48 |  |
| SENP8_HUMAN | 5.43 | 1xt9 |
| SERC_HUMAN | 5.88 |  |
| SF3B4_HUMAN | 5.02 |  |
| SFRS4_HUMAN | 5.28 |  |
| SFRS7_HUMAN | 5.13 | 2hvz |
| SHC1_HUMAN | 6.53 | 1shc |
| SHLB2_HUMAN | 5.09 |  |
| SIAS_HUMAN | 5.47 | 1wvo |
| SIL1_HUMAN | 1.53 |  |
| SMAD4_HUMAN | 6.30 |  |
| SMR3B_HUMAN | 1.64 |  |
| SNRPA_HUMAN | 5.45 | 1oia |
| SNURF_HUMAN | 5.71 |  |
| SNX16_HUMAN | 5.57 |  |
| SNX17_HUMAN | 5.32 | 3lui |
| SNX9_HUMAN | 5.50 | 2rak |
| SODC_HUMAN | 5.60 | 3ecu |
| SPB6_HUMAN | 5.46 |  |
| SPB9_HUMAN | 6.13 |  |
| SPI2_HUMAN | 1.77 |  |
| SPIR1_HUMAN | 1.97 |  |
| SPOP_HUMAN | 1.75 | 3ivv |
| SPS1_HUMAN | 5.65 | 3fd6 |
| SSA27_HUMAN | 5.17 |  |
| SSBP3_HUMAN | 5.15 |  |
| ST2B1_HUMAN | 5.92 | 1q22 |
| ST32B_HUMAN | 5.03 |  |
| STIP1_HUMAN | 5.93 | 1elr |
| STMN1_HUMAN | 6.31 |  |
| STRAP_HUMAN | 5.22 |  |
| STUB1_HUMAN | 5.20 |  |
| SUMO2_HUMAN | 6.12 | 2awt |
| SUMO3_HUMAN | 5.46 | 2io1 |
| SURF2_HUMAN | 5.13 |  |
| SUV91_HUMAN | 5.22 |  |
| SYAP1_HUMAN | 5.15 | 1x3a |
| SYNC_HUMAN | 6.06 |  |
| SYTC_HUMAN | 5.29 | 1wwt |
| SYUG_HUMAN | 5.94 |  |
| SYWC_HUMAN | 5.66 | 2quk |
| TALDO_HUMAN | 5.13 | 1f05 |
| TBB3_HUMAN | 5.05 |  |
| TBB4_HUMAN | 5.05 |  |
| TBB5_HUMAN | 5.19 |  |
| TBB6_HUMAN | 5.04 |  |
| TBCC_HUMAN | 5.33 | 2yuh |
| TBCE_HUMAN | 5.81 |  |
| TBE_HUMAN | 5.19 |  |
| TBRG1_HUMAN | 1.66 |  |
| TCTA_HUMAN | 1.69 |  |
| TEBP_HUMAN | 5.77 | 1ejf |
| TETN_HUMAN | 1.84 | 1tn3 |
| TEX12_HUMAN | 1.83 |  |
| TFF1_HUMAN | 1.75 | 1hi7 |
| TFF2_HUMAN | 1.59 |  |
| TFG_HUMAN | 5.14 |  |
| TFIP8_HUMAN | 5.13 |  |
| TFPI1_HUMAN | 1.78 | 1adz |
| TGFA1_HUMAN | 5.11 |  |
| THOC3_HUMAN | 5.12 |  |
| THOP1_HUMAN | 5.36 |  |
| THTPA_HUMAN | 6.07 |  |
| THUM1_HUMAN | 5.73 | 2dir |
| THUM3_HUMAN | 5.13 |  |
| TKN1_HUMAN | 1.71 | 2b19 |
| TNF13_HUMAN | 1.88 |  |
| TPD52_HUMAN | 5.54 |  |
| TPD54_HUMAN | 6.07 |  |
| TPIS_HUMAN | 6.44 | 2jk2 |
| TRBP2_HUMAN | 5.36 | 2cpn |
| TRI38_HUMAN | 5.57 |  |
| TSG6_HUMAN | 1.75 | 2pf5 |
| TTC19_HUMAN | 1.88 |  |
| TTC4_HUMAN | 5.67 |  |
| TXD12_HUMAN | 1.86 | 1sen |
| TXND3_HUMAN | 5.94 |  |
| TYB10_HUMAN | 5.90 |  |
| U2AF2_HUMAN | 5.08 | 1u2f |
| UAP1_HUMAN | 6.00 | 1jvg |
| UB2Q1_HUMAN | 1.83 | 2qgx |
| UB2Q2_HUMAN | 1.86 |  |
| UB2R2_HUMAN | 1.69 |  |
| UBE2U_HUMAN | 1.58 | 1yrv |
| UBP12_HUMAN | 1.92 |  |
| UCHL1_HUMAN | 5.75 |  |
| UCHL3_HUMAN | 6.11 | 1xd3 |
| UFM1_HUMAN | 5.26 | 1wxs |
| UROK_HUMAN | 2.00 | 3ig6 |
| UTP11_HUMAN | 1.56 |  |
| VIME_HUMAN | 5.16 | 1gk4 |
| VINEX_HUMAN | 5.49 | 2ct3 |
| WD51A_HUMAN | 5.41 |  |
| WDR33_HUMAN | 1.70 |  |
| WDR5_HUMAN | 5.52 | 2gnq |
| WDR62_HUMAN | 5.40 |  |
| WIBG_HUMAN | 5.82 |  |
| WIF1_HUMAN | 1.72 |  |
| WWP2_HUMAN | 5.34 |  |
| XRCC4_HUMAN | 5.39 |  |
| ZBP1_HUMAN | 1.93 | 3eyi |
| ZC3HA_HUMAN | 5.38 |  |
| ZCH10_HUMAN | 5.08 |  |
| ZCH12_HUMAN | 1.90 |  |
| ZCH13_HUMAN | 5.62 |  |
| ZG16_HUMAN | 1.79 |  |
| ZMAT5_HUMAN | 1.84 |  |
| ZMY10_HUMAN | 5.33 | 2d8q |
| ZN616_HUMAN | 1.69 |  |
| ZNF18_HUMAN | 5.06 |  |
| ZNF85_HUMAN | 1.82 |  |

**Table 4: Overview of the chaperone set. This table contains the chaperones used in the IntAct [4] interaction study, represented by their accession number, entry name and UniProt comment.**

| **sHSP** | | |
| --- | --- | --- |
| **Accession** | **Entry name** | **Uniprot comments** |
| P04792 | HSPB1_HUMAN | Heat shock protein beta-1 (HspB1) (Heat shock 27 kDa protein) (HSP 27) (Stress-responsive protein 27) (SRP27) (Estrogen-regulated 24 kDa protein) (28 kDa heat shock protein) |
| A8KAH6 | A8KAH6_HUMAN | Heat shock 27kDa protein 2, isoform CRA_a (Heat-shock protein beta-2) (cDNA FLJ77220, highly similar to Homo sapiens heat shock 27kDa protein 2 (HSPB2), mRNA) |
| Q6ICS9 | Q6ICS9_HUMAN | HSPB3 protein (Heat shock 27kDa protein 3) (cDNA, FLJ94950, Homo sapiens heat shock 27kDa protein 3 (HSPB3), mRNA) (Fragment) |
| P02489 | CRYAA_HUMAN | Alpha-crystallin A chain (Heat shock protein beta-4) (HspB4) [Cleaved into: Alpha-crystallin A chain, short form] |
| P02511 | CRYAB_HUMAN | Alpha-crystallin B chain (Alpha(B)-crystallin) (Rosenthal fiber component) (Heat shock protein beta-5) (HspB5) (Renal carcinoma antigen NY-REN-27) |
| O14558 | HSPB6_HUMAN | Heat shock protein beta-6 (HspB6) (Heat shock 20 kDa-like protein p20) |
| Q9UBY9 | HSPB7_HUMAN | Heat shock protein beta-7 (HspB7) (Cardiovascular heat shock protein) (cvHsp) |
| Q9UJY1 | HSPB8_HUMAN | Heat shock protein beta-8 (HspB8) (Alpha-crystallin C chain) (Small stress protein-like protein HSP22) (E2-induced gene 1 protein) (Protein kinase H11) |
| Q9BQS6 | HSPB9_HUMAN | Heat shock protein beta-9 (HspB9) (Cancer/testis antigen 51) (CT51) |
| Q9Y547 | HSB11_HUMAN | Heat shock protein beta-11 (Hspb11) (Placental protein 25) (PP25) |

| **Hsp40** |  |  |
| --- | --- | --- |
| **Accession** | **Entry** | **Uniprot comments** |
| P31689 | DNJA1_HUMAN | DnaJ homolog subfamily A member 1 (Heat shock 40 kDa protein 4) (DnaJ protein homolog 2) (HDJ-2) (HSJ-2) (HSDJ) |
| O60884 | DNJA2_HUMAN | DnaJ homolog subfamily A member 2 (HIRA-interacting protein 4) (Cell cycle progression restoration gene 3 protein) (Dnj3) (Dj3) (Renal carcinoma antigen NY-REN-14) |
| Q96EY1 | DNJA3_HUMAN | DnaJ homolog subfamily A member 3, mitochondrial (Tumorous imaginal discs protein Tid56 homolog) (DnaJ protein Tid-1) (hTid-1) (Hepatocellular carcinoma-associated antigen 57) |
| Q6AW87 | Q6AW87_HUMAN | Putative uncharacterized protein DKFZp686G2074 (DnaJ (Hsp40) homolog, subfamily A, member 4, isoform CRA_b) |
| Q5F1R6 | DJC21_HUMAN | DnaJ homolog subfamily C member 21 (DnaJ homolog subfamily A member 5) (Protein GS3) |
| P25685 | DNJB1_HUMAN | DnaJ homolog subfamily B member 1 (Heat shock 40 kDa protein 1) (Heat shock protein 40) (HSP40) (DnaJ protein homolog 1) (HDJ-1) |
| P25686 | DNJB2_HUMAN | DnaJ homolog subfamily B member 2 (Heat shock 40 kDa protein 3) (DnaJ protein homolog 1) (HSJ-1) |
| Q9UDY4 | DNJB4_HUMAN | DnaJ homolog subfamily B member 4 (Heat shock 40 kDa protein 1 homolog) (Heat shock protein 40 homolog) (HSP40 homolog) (Human liver DnaJ-like protein) |
| O75953 | DNJB5_HUMAN | DnaJ homolog subfamily B member 5 (Heat shock protein Hsp40-3) (Heat shock protein cognate 40) (Hsc40) (Hsp40-2) |
| O75190 | DNJB6_HUMAN | DnaJ homolog subfamily B member 6 (Heat shock protein J2) (HSJ-2) (MSJ-1) (HHDJ1) (MRJ) |
| Q7Z6W7 | DNJB7_HUMAN | DnaJ homolog subfamily B member 7 |
| Q8NHS0 | DNJB8_HUMAN | DnaJ homolog subfamily B member 8 |
| Q9UBS3 | DNJB9_HUMAN | DnaJ homolog subfamily B member 9 (Microvascular endothelial differentiation gene 1 protein) (Mdg-1) |
| Q9UBS4 | DJB11_HUMAN | DnaJ homolog subfamily B member 11 (ER-associated dnaJ protein 3) (ERj3p) (ERdj3) (ER-associated Hsp40 co-chaperone) (ER-associated DNAJ) (HEDJ) (hDj9) (PWP1-interacting protein 4) (APOBEC1-binding protein 2) (ABBP-2) |
| Q9NXW2 | DJB12_HUMAN | DnaJ homolog subfamily B member 12 |
| Q8TBM8 | DJB14_HUMAN | DnaJ homolog subfamily B member 14 |
| Q5T1X3 | Q5T1X3_HUMAN | DnaJ (Hsp40) homolog, subfamily C, member 1 (Fragment) |
| Q99543 | DNJC2_HUMAN | DnaJ homolog subfamily C member 2 (Zuotin-related factor 1) (M-phase phosphoprotein 11) |
| Q13217 | DNJC3_HUMAN | DnaJ homolog subfamily C member 3 (Interferon-induced, double-stranded RNA-activated protein kinase inhibitor) (Protein kinase inhibitor of 58 kDa) (Protein kinase inhibitor p58) |
| Q9NNZ3 | DNJC4_HUMAN | DnaJ homolog subfamily C member 4 (Multiple endocrine neoplasia type 1 candidate protein number 18) (DnaJ-like protein HSPF2) |
| Q9H3Z4 | DNJC5_HUMAN | DnaJ homolog subfamily C member 5 (Cysteine string protein) (CSP) |
| Q9UF47 | DNJ5B_HUMAN | DnaJ homolog subfamily C member 5B (Beta cysteine string protein) (Beta-CSP) |
| Q8N7S2 | DNJ5G_HUMAN | DnaJ homolog subfamily C member 5G (Gamma-cysteine string protein) (Gamma-CSP) |
| O75061 | AUXI_HUMAN | Putative tyrosine-protein phosphatase auxilin (EC 3.1.3.48) (DnaJ homolog subfamily C member 6) |
| Q99615 | DNJC7_HUMAN | DnaJ homolog subfamily C member 7 (Tetratricopeptide repeat protein 2) (TPR repeat protein 2) |
| O75937 | DNJC8_HUMAN | DnaJ homolog subfamily C member 8 (Splicing protein spf31) |
| Q8WXX5 | DNJC9_HUMAN | DnaJ homolog subfamily C member 9 (DnaJ protein SB73) |
| Q8IXB1 | DJC10_HUMAN | DnaJ homolog subfamily C member 10 (ER-resident protein ERdj5) (Macrothioredoxin) (MTHr) |
| Q9NVH1 | DJC11_HUMAN | DnaJ homolog subfamily C member 11 |
| Q5JVQ1 | Q5JVQ1_HUMAN | J domain containing protein 1 (JDP1) (DnaJ (Hsp40) homolog, subfamily C, member 12, isoform CRA_c) |
| O75165 | DJC13_HUMAN | DnaJ homolog subfamily C member 13 (Required for receptor-mediated endocytosis 8) (RME-8) |
| Q6Y2X3 | DJC14_HUMAN | DnaJ homolog subfamily C member 14 (Dopamine receptor-interacting protein of 78 kDa) (DRiP78) (DnaJ protein homolog 3) (HDJ-3) |
| Q9Y5T4 | DJC15_HUMAN | DnaJ homolog subfamily C member 15 (Methylation-controlled J protein) (MCJ) (Cell growth-inhibiting gene 22 protein) |
| Q5TDH4 | Q5TDH4_HUMAN | DnaJ (Hsp40) homolog, subfamily C, member 16 |
| Q9NVM6 | DJC17_HUMAN | DnaJ homolog subfamily C member 17 |
| Q9H819 | DJC18_HUMAN | DnaJ homolog subfamily C member 18 |
| Q96DA6 | TIM14_HUMAN | Mitochondrial import inner membrane translocase subunit TIM14 (DnaJ homolog subfamily C member 19) |
| Q8IWL3 | HSC20_HUMAN | Co-chaperone protein HscB, mitochondrial (DnaJ homolog subfamily C member 20) (Hsc20) |
| Q5F1R6 | DJC21_HUMAN | DnaJ homolog subfamily C member 21 (DnaJ homolog subfamily A member 5) (Protein GS3) |
| Q8N4W6 | DJC22_HUMAN | DnaJ homolog subfamily C member 22 |
| Q9H1X3 | DJC25_HUMAN | DnaJ homolog subfamily C member 25 |
| Q9NX36 | DJC28_HUMAN | DnaJ homolog subfamily C member 28 |

| **Hsp70** |  |  |
| --- | --- | --- |
| **Accesion** | **Entry_name** | **Uniprot comments** |
| P08107 | HSP71_HUMAN | Heat shock 70 kDa protein 1 (HSP70.1) (HSP70-1/HSP70-2) |
| P34931 | HS71L_HUMAN | Heat shock 70 kDa protein 1L (Heat shock 70 kDa protein 1-like) (Heat shock 70 kDa protein 1-Hom) (HSP70-Hom) |
| P54652 | HSP72_HUMAN | Heat shock-related 70 kDa protein 2 (Heat shock 70 kDa protein 2) |
| P34932 | HSP74_HUMAN | Heat shock 70 kDa protein 4 (Heat shock 70-related protein APG-2) (HSP70RY) |
| P11021 | GRP78_HUMAN | 78 kDa glucose-regulated protein (GRP 78) (Heat shock 70 kDa protein 5) (Immunoglobulin heavy chain-binding protein) (BiP) (Endoplasmic reticulum lumenal Ca(2+)-binding protein grp78) |
| P17066 | HSP76_HUMAN | Heat shock 70 kDa protein 6 (Heat shock 70 kDa protein B') |
| P48741 | HSP77_HUMAN | Putative heat shock 70 kDa protein 7 (Heat shock 70 kDa protein B) |
| P11142 | HSP7C_HUMAN | Heat shock cognate 71 kDa protein (Heat shock 70 kDa protein 8) |
| P38646 | GRP75_HUMAN | Stress-70 protein, mitochondrial (75 kDa glucose-regulated protein) (GRP 75) (Heat shock 70 kDa protein 9) (Peptide-binding protein 74) (PBP74) (Mortalin) (MOT) |
| P48723 | HSP13_HUMAN | Heat shock 70 kDa protein 13 (Stress 70 protein chaperone microsome-associated 60 kDa protein) (Microsomal stress 70 protein ATPase core) |

| **Hsp90** |  |  |
| --- | --- | --- |
| **Accession** | **Entry** | **Uniprot comments** |
| P07900 | HS90A_HUMAN | Heat shock protein HSP 90-alpha (HSP 86) (Renal carcinoma antigen NY-REN-38) |
| Q14568 | HS902_HUMAN | Putative heat shock protein HSP 90-alpha A2 (Heat shock 90 kDa protein 1 alpha-like 3) |
| Q58FG1 | HS904_HUMAN | Putative heat shock protein HSP 90-alpha A4 (Heat shock protein 90-alpha D) (Heat shock protein 90Ad) (Heat shock 90 kDa protein 1 alpha-like 2) |
| Q58FG0 | HS905_HUMAN | Putative heat shock protein HSP 90-alpha A5 (Heat shock protein 90-alpha E) (Heat shock protein 90Ae) |
| O75322 | O75322_HUMAN | Raf activator, no geldanamycin binding region, overexpressed in pancreatic carcinomas |
| P08238 | HS90B_HUMAN | Heat shock protein HSP 90-beta (HSP 90) (HSP 84) |
| Q58FF8 | H90B2_HUMAN | Putative heat shock protein HSP 90-beta 2 (Heat shock protein 90-beta b) (Heat shock protein 90Bb) |
| Q58FF7 | H90B3_HUMAN | Putative heat shock protein HSP 90-beta-3 (Heat shock protein 90-beta c) (Heat shock protein 90Bc) |
| Q58FF6 | H90B4_HUMAN | Putative heat shock protein HSP 90-beta 4 |
| P14625 | ENPL_HUMAN | Endoplasmin (Heat shock protein 90 kDa beta member 1) (94 kDa glucose-regulated protein) (GRP94) (gp96 homolog) (Tumor rejection antigen 1) |
| Q96GW1 | Q96GW1_HUMAN | HSP90B1 protein |
| Q58FF3 | ENPLL_HUMAN | Putative endoplasmin-like protein (Putative heat shock protein 90 kDa beta member 2) |
| Q58FF2 | Q58FF2_HUMAN | Heat shock protein 94c |
|  |  |  |
| Q12931 | TRAP1_HUMAN | Heat shock protein 75 kDa, mitochondrial (HSP 75) (Tumor necrosis factor type 1 receptor-associated protein) (TRAP-1) (TNFR-associated protein 1) |

1. Yen HC, Xu Q, Chou DM, Zhao Z, Elledge SJ (2008) Global protein stability profiling in mammalian cells. Science 322: 918-923.

2. Ghersi-Egea JF, Gorevic PD, Ghiso J, Frangione B, Patlak CS, et al. (1996) Fate of cerebrospinal fluid-borne amyloid beta-peptide: rapid clearance into blood and appreciable accumulation by cerebral arteries. J Neurochem 67: 880-883.

3. Berman HM, Westbrook J, Feng Z, Gilliland G, Bhat TN, et al. (2000) The Protein Data Bank. Nucleic Acids Res 28: 235-242.

4. Aranda B, Achuthan P, Alam-Faruque Y, Armean I, Bridge A, et al. (2010) The IntAct molecular interaction database in 2010. Nucleic Acids Res 38: D525-531.
